# Binding Gel Optimization for the Detection of Organomineral Iron Colloids with the Diffusive Gradients in Thin Films Method

**DOI:** 10.1021/acs.analchem.5c02501

**Published:** 2025-11-24

**Authors:** Jasnalien Ceulemans, Claudia Moens, Erik Smolders

**Affiliations:** Division of Soil and Water Management, Department of Earth and Environmental Sciences, 26657 KU Leuven, Kasteelpark Arenberg 20, 3001 Heverlee, Belgium

## Abstract

Mobile soil colloids are reactive but are difficult to sample from soil in an unbiased way. The use of the diffusive gradients in thin films (DGT) technique is proposed for *in situ* colloid sampling in undisturbed soil. Here, the first step is presented by the development of a DGT binding layer that enables the accumulation of organomineral iron (Fe) colloids. A high sorption capacity and a high affinity of organic anion binding sites are required to ensure their detection on the gel. The existing 0.1 M ZrO_2_ binding gel of *in situ* precipitated ZrOCl_2_ was suggested as a candidate and validated for organomineral Fe colloid accumulation. It was speculated that higher colloid capacity and affinity might be achieved by enhanced concentrations and smaller dimensions of the metal oxide adsorbent particles, which can be obtained by changing the precursor concentration and type. Increasing the ZrOCl_2_ concentration had the largest effect on organomineral Fe colloid and phosphate accumulations. Alternative ZrCl_4_ and Zr­(OC_4_H_9_)_4_ precursors or TiO_2_ and Nb_2_O_5_ adsorbents did neither distinctly enhance the sorption capacity nor the colloid affinity. Incorporating the polyvinylpyrrolidone adsorbent within the hydrogel matrix only marginally enhanced organomineral Fe colloid sorption. Finally, an *in situ* precipitated, 0.2 M ZrOCl_2_-based binding gel with superior sorption properties is put forward as the most promising binding layer for *in situ* organomineral Fe colloid detection in soil.

## Graphical abstract

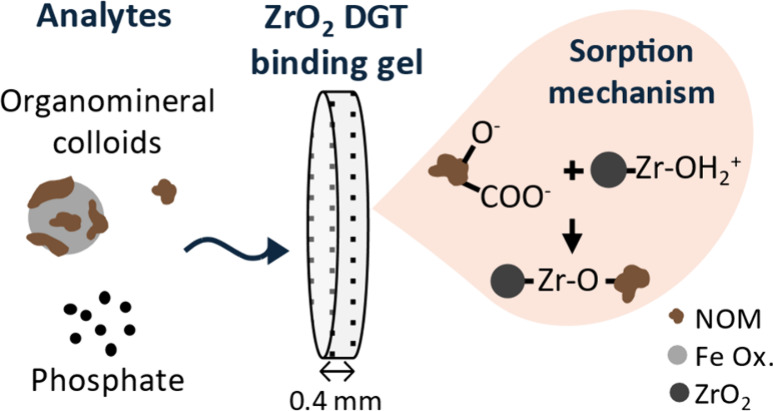

Natural soil colloids can have a substantial role in nutrient and contaminant migration and bioavailability. The principal colloid types in soil pore water comprise particles of natural organic matter (NOM), iron (Fe) and aluminum oxyhydroxides, and clay minerals with one dimension smaller than 1 μm. Their small size and large specific surface area make them effective carriers of nutrients and contaminants in soil, including oxyanions, trace metals, and radionuclides.^1,2^ Soil colloids can facilitate leaching, e.g., by associations between phosphate (PO_4_) and oxyhydroxides,^3^ cadmium and NOM,^4^ or radiocaesium and clays.^2^ Colloidal phosphorus (P) can contribute to plant P uptake.^5^ However, sampling artifacts hamper the understanding of colloid significance in this mobility and plant availability. Traditional soil solution sampling methods lead to biases in measured colloid concentrations caused by changes in the *in situ* ionic strength in soil extraction protocols, disruption of soil structure in soil centrifugation protocols, or trapping of colloids onto filters.^4,6,7^ Hence, there is a need for an *in situ* method that samples soil colloids with less disturbance.

The diffusive gradients in thin films (DGT) technique allows *in situ* sampling of labile species in soil. The method was originally developed by Davison and Zhang for *in situ* measurement of trace metal ion concentrations in natural waters.^8^ The DGT setup consists of a stack of an adsorbent-containing hydrogel binding layer, a hydrogel diffusion layer, and a protective membrane. Solutes accumulate in the zero-sink binding gel after mobilization by controlled diffusion from the bulk solution through the diffusion layer. The time-averaged concentration at the interface of DGT and solution (*c*_DGT_) can be calculated by measuring the accumulated analytes.^9^ The DGT application has been extended for analysis of many other solutes and for deployment in sediments and soil,^10^ where analytes both present in the pore water and buffered by sorption to particles determine the *c*_DGT_.^11^ Moreover, the DGT technique can be used for high-resolution imaging of DGT-labile elements in soil and sediments.^12^ The binding layer is then analyzed with a spatially resolved method such as laser ablation inductively coupled plasma mass spectrometry (LA-ICP-MS).^13^

The DGT has the potential to not only sample solutes but also mobile natural colloids *in situ* with limited soil disturbance. To this end, colloids need to permeate the diffusion layer and accumulate in the binding layer. Colloid diffusivity in hydrogels is still unclear, but synthetic particles up to 130 nm radius have been shown to substantially permeate agarose derivative-cross-linked polyacrylamide (APA) hydrogels that are common diffusive and binding gels.^14^ Natural colloidal species have been measured with solute-specific DGTs, such as NOM-complexed metals.^15^ Targeted analysis of manufactured 30–50 nm zinc oxide colloids in soil was achieved, for which standard DGTs were compared with modified DGTs that exclude colloids by means of a dialysis membrane.^16^ Differentiation between solutes and colloidal species in DGTs has also been realized by time-resolved analysis or colloid exclusion based on diffusion rate differences.^17,18^ The substantially smaller diffusion coefficients of colloids than solutes result in lower sensitivities in DGTs.^9^ Nevertheless, it has been shown that colloidal P can contribute to the P DGT flux when DGTs were deployed on a substrate with extracted soil colloids, and in nonfiltered versus ultrafiltered soil extracts.^5,19^ As far as is known, no binding layer has yet been specifically developed as part of the DGT method to analyze the major natural soil colloids quantitatively.

The most common colloidal Fe oxyhydroxides are predominantly negatively charged in pore water due to associations with NOM.^2^ Therefore, they are expected to be sorbed in binding layers used for anionic species, by adsorbents such as zirconium oxide (ZrO_2_)^20^ or titanium oxide-based (TiO_2_) Metsorb.^21^ It is hypothesized that organomineral Fe colloids can be accumulated via interactions with negatively charged carboxylate and phenolate reactive groups of the NOM with the metal oxides. This colloid sorption, however, can be inhibited by the competition effects of pore water anions, predominantly PO_4_.^22^ Colloids are disadvantaged by their much lower diffusion rates toward the sorption sites than solutes.^9^

The aim of this study was to optimize a binding layer for organomineral Fe colloids. Different original approaches were tested to create a binding layer for organomineral Fe with high sorption capacity and organomineral Fe colloid affinity. The starting point was a 0.1 M ZrO_2_ binding layer of ZrOCl_2_ precipitated in a hydrogel,^23^ which is commercially available (DGT Research Ltd.). This binding gel was selected because *in situ* ZrO_2_ precipitation generates homogeneously dispersed and small-sized (<0.2 μm) adsorbents, in contrast with the incorporation of presynthesized ZrO_2_ particles.^20,23^ Moreover, it has been validated for high-resolution oxyanion imaging.^23^ A first approach to optimize this binding gel for organomineral Fe colloids was to increase the sorption capacity by obtaining smaller ZrO_2_ particles with larger reactive surface areas. This size reduction could also enhance the selectivity of colloids over competing anions since a size-dependent NOM-PO_4_ competition, presumably due to steric interactions, has been observed.^24^ To this end, two precursors other than the prevalent ZrOCl_2_ were explored for *in situ* ZrO_2_ precipitation. The ZrCl_4_ precursor induces more acid conditions by hydrolysis than ZrOCl_2_, which potentially enhances hydrolysis product dispersion and limits polymerization, resulting in smaller ZrO_2_ particles.^25^ The Zr­(OC_4_H_9_)_4_ precursor may lead to smaller ZrO_2_ particles through fast hydrolysis reactions^26^ and due to steric hindrance in the polymerization reaction by the organic tails. A second approach was to augment the sorption capacity by increasing the ZrOCl_2_ concentration, thus the binding gel ZrO_2_ content, as previously suggested.^23^ A third approach was to increase the NOM affinity by *in situ* precipitation of other metal oxides than ZrO_2_. It has been shown that TiO_2_ can adsorb humic acids by their phenolate groups.^27^ Mixing the commercially available Metsorb within gel solutions before casting successfully introduced TiO_2_ in DGTs for measuring arsenic, selenium and P oxyanions.^21,28^ *In situ* TiO_2_ precipitation, however, has not yet been tested in DGT applications. Furthermore, niobium oxide (Nb_2_O_5_) has been shown to have a strong affinity for carboxylates, even in the presence of competing molecules,^29^ but has not yet been examined as a DGT adsorbent. A last approach for optimizing organomineral Fe binding layers was to enhance the NOM affinity by adapting the hydrogel itself through the incorporation of the nonionic polymeric polyvinylpyrrolidone (PVP) adsorbent. Polymer-based resins such as XAD18 and hydrophilic–lipophilic balance (HLB) have been used as binding layer adsorbents in DGTs developed for organic compound sampling (o-DGT).^30,31^ The PVP can accumulate NOM via hydrogen bonding between aromatic hydroxyl and carboxyl groups.^32^ Mixing of PVP within the APA gel matrix, which has not been done yet in DGT applications, generates semi-interpenetrating polymer network hydrogels^33,34^ and potentially allows cooperative NOM binding with metal oxides. The PVP incorporation was tested with hydrogels of both agarose and APA because the larger pore sizes of agarose compared to APA hydrogels were expected to be advantageous for colloid permeation.^9,35^

Taken together, the DGT technique has the potential to sample natural soil colloids *in situ* and map their distribution in soil at micrometre resolution. This work was set up to develop binding layers able to bind organomineral Fe colloids. It presents a comparison of newly synthesized binding gels, with a focus on metal oxide adsorbent properties and hydrogel characteristics for enhancing sorption capacity and colloid affinity. The tested organomineral Fe colloids are lab-synthesized (i) Fe-NOM complexes (2 nm) to verify the hypothesized NOM sorption with detection based on Fe, and (ii) NOM-coated hydrous ferric oxide (HFO) colloids (50 nm) to verify binding gel permeability of larger colloids. Colloid Fe detection on binding layers was verified using dialysis membranes. Total PO_4_ sorption capacities were also determined as a probe for the reactive surface area of the metal oxides in the binding gels. Finally, the best-performing binding gel was validated in terms of linearity in sorption as a function of time and colloid concentration.

## Experimental Section

Several types of binding layers were synthesized by *in situ* metal oxide precipitation in a hydrogel, for which PO_4_ and organomineral Fe colloid sorption properties were tested (Supporting Information, Table S1). Gel discs of a commercially available ZrO_2_ APA gel sheet (R-GSZR, DGT Research Ltd.) were always included as a reference in sorption tests and digestions (average measured Zr concentration of 0.08 ± 0.01 M).

### Binding Gel Synthesis

#### *In Situ* Precipitated ZrO_2_, TiO_2_, and Nb_2_O_5_ Gels

Hydrogels of APA were prepared according to standard procedures (S1.1.)^36,37^ for *in situ* metal oxide precipitation based on Guan *et al*.^23^ The ZrOCl_2_ (ZrOCl_2_.xH_2_O) precursor was dissolved in ultrapure (Milli-Q) water at a 0.1 M concentration in a total volume of 33 mL, including a submerged APA gel sheet (23 cm × 7 cm × 0.04 cm). Similarly, gel-containing solutions were prepared with up to 1 M ZrOCl_2_, and with nominal concentrations of 0.1 and 0.3 M ZrCl_4_ and TiCl_4_. The NbCl_5_ and n-butoxide precursors, Zr­(OC_4_H_9_)_4_ (80% in butanol), Ti­(OC_4_H_9_)_4_, and Nb­(OC_4_H_9_)_5_, were dissolved in 12 M hydrochloric acid (HCl) to slow down precipitation (S1.2.). These solutions were diluted with Milli-Q water to nominal concentrations of 0.1 and 0.3 M in 5 M HCl, with APA gels presoaked in 5 M HCl. Hereafter, each gel-containing metal solution was placed on a horizontal shaker to equilibrate for 2 h. The 0.1 M ZrOCl_2_ gel was then transferred to 100 mL of a 0.05 M 2-(N-morpholino)­ethanesulfonic acid (MES) buffer solution at pH 6.7 for ZrO_2_ precipitation. Meanwhile, the other gels were submerged in adapted volumes of 0.05 and 0.5 M MES solutions to buffer the proton concentrations expected from the precursors’ hydrolysis reactions and added HCl (S1.2.). All buffer solutions were horizontally shaken for 45 min to complete *in situ* ZrO_2_, TiO_2_ or Nb_2_O_5_ precipitation and their pH was verified to be >6. Finally, the binding gels were washed for 24 h in Milli-Q water and stored in 10 mM sodium chloride (NaCl) solutions.

#### Hybrid PVP Hydrogels

Semi-interpenetrating polymer network or hybrid gels were synthesized by incorporating PVP into the APA matrix, based on Evingür *et al*.^38^ A hydrogel PVP content of 1.5% m/v was selected after a preliminary NOM sorption test with 0.3–6% PVP gels (Figure S1). Standard APA gel solution preparation and casting were performed with 10% PVP (average molecular weight 40,000) dissolved in the Milli-Q water to obtain a 1.5% PVP hybrid hydrogel after hydration. Accordingly, PVP was integrated into agarose hydrogels (S1.1.) by mixing 1.5% PVP with the agarose and Milli-Q water before heating and gel solution casting. *In situ* ZrO_2_ precipitation with ZrOCl_2_ was done in the APA- and agarose-PVP gels as previously described.

### Sorption Tests in Synthetic Solutions

#### Total PO_4_ Sorption Capacity

The maximal PO_4_ accumulation in a binding gel, termed the total PO_4_ sorption capacity (μg P cm^–2^), was determined as an indicator of its overall analyte sorption capacity. Moreover, it reflects the reactive surface area of the metal oxide adsorbents. This total capacity is to be distinguished from the DGT capacity commonly defined in DGT literature, i.e., the binding gel PO_4_ content up to which PO_4_ uptake linearly increases with increasing PO_4_ concentration in the bulk solution. A highly concentrated KH_2_PO_4_ solution (400 mg P L^–1^) was prepared at pH 6 with 10 mM NaCl as background electrolyte. Binding gel discs (*n* = 2, *r* = 1.2 cm) were individually deployed in 250 mL PO_4_ solution for 24 h at a constant temperature of 20 °C to eliminate the influence of temperature changes on analyte diffusion and adsorption rates. Depletion of PO_4_ in the deployment solutions was <1.5%.

#### Organomineral Fe Colloid Sorption Affinity

The binding layers were tested for uptake of two organomineral Fe colloid types, mononuclear Fe-NOM complexes (2 nm, referred to as Fe-NOM) and larger NOM-coated hydrous ferric oxide particles (50 nm, referred to as HFO). The colloids were freshly synthesized by Fe­(II) oxidation in the presence of Suwannee River NOM (SRNOM, 2R101N, International Humic Substances Society) according to the protocol of Moens and Smolders.^24^ The preparation was verified by flow field-flow fractionation coupled to ICP-MS, a technique by which the model colloids were previously characterized (Figure S2 and S1.3.).^24^ The colloid suspensions nominally contained 5 mg Fe L^–1^ in 10 mM NaCl at pH 6, with SRNOM at an organic carbon (OC) over Fe molar ratio of 110 for Fe-NOM and 2 for HFO. Binding gel discs were placed in plastic DGT housings for solution deployment (*n* = 2, *r* = 1 cm), with a supportive hydrogel underneath to fill the space of the omitted diffusive gel and filter membrane. They were deployed together in a well-stirred suspension for exposure to an equal bulk colloid concentration, with a total volume equivalent to 50 mL per disc for 24 h at 20 °C. Organomineral Fe colloid uptake in the binding gels was compared by measuring Fe as a proxy. The suspensions during tests for the effect of either ZrO_2_ concentration and metal oxide type or hydrogel type were depleted <14% in Fe.

The origin of Fe detected on binding layers was elucidated by differentiation between truly dissolved and colloidal species using a 1 kDa molecular weight cutoff (MWCO) dialysis membrane (Spectra/Por 7, Spectrum Laboratories), as has been done before in DGTs.^16^ Binding gel discs of 0.2 M ZrOCl_2_-based gels (0.24 M Zr measured) were placed in DGT housings (*n* = 2, *r* = 1 cm) either without a diffusion layer or with a dialysis membrane in front. They were deployed together in well-stirred Fe-NOM and HFO suspensions with a total volume equivalent to 50 mL per disc for 24 h at 20 °C. A dialysis membrane with 2.5 mL of 10 mM NaCl solution was simultaneously deployed with the binding gels to determine the truly dissolved Fe fractions in the colloid suspensions. Depletions of Fe in the Fe-NOM and HFO suspensions were 9% and 23%, whereas the truly dissolved Fe concentrations were 0.5 and 0.8 mg Fe L^–1^. Smaller discs (*r* = 0.85 cm) were cut out of the exposed binding gel areas for analysis to avoid potential contamination of colloids that might have reached the binding gel by passing in between the base and cap of the DGT housing.

#### Time- and Concentration-Dependent Fe-NOM Sorption

The sorption of Fe-NOM colloids as a function of time and Fe-NOM concentration was tested with 0.1 and 0.2 M ZrOCl_2_-based binding gels. First, a Fe-NOM suspension (see previous section) was diluted to 0.5 mg Fe L^–1^ in 10 mM NaCl at pH 6. Binding gel discs (*n* = 2, *r* = 1.2 cm) were deployed together in 2.1 L of well-stirred Fe-NOM suspension at 20 °C and removed at 14 different times between 1 min and 24 h. The suspensions at the last time point were depleted by 12% and 7% in Fe due to the 0.1 and 0.2 M Zr gels, respectively. Second, a Fe-NOM suspension was diluted in 10 mM NaCl at pH 6 to obtain a concentration range of 0.05–5 mg Fe L^–1^. Binding gel discs were placed in DGT housings without diffusion layers (*n* = 2, *r* = 1 cm) and individually deployed in 50 mL of well-stirred Fe-NOM suspension for 24 h at 20 °C. The maximal Fe depletion in the suspensions decreased with increasing Fe concentration from 66% to 7% for the 0.1 M Zr gels and from 58% to 11% for the 0.2 M Zr gels.

### Elemental Analysis

Binding layer elution, typically done for the quantification of accumulated analytes, is feasible for 0.1 M ZrOCl_2_-based gels with sodium hydroxide (NaOH) for PO_4_ ^23^ and Fe-NOM colloids (S1.4. and Table S2). Nevertheless, binding gel discs were digested to simultaneously determine the content of *in situ* precipitated metal and accumulated target analyte. Digestion was performed in a microwave system (Mars 6, CEM) based on a previously described protocol for metal oxide DGT binding layers.^39^ Deployed gel discs were rinsed with Milli-Q water, air-dried, and submerged in 2 mL of nitric acid (HNO_3_, 70%) and 4 mL of sulfuric acid (H_2_SO_4_, 95%). Thereafter followed an overnight predigestion at room temperature and a consecutive microwave-assisted 100 °C predigestion and 30 min 210 °C digestion. Generally, a near-total ZrO_2_ destruction is accomplished by a hot plate four-acid digestion, using HNO_3_, hydrofluoric acid (HF), perchloric acid (HClO_4_), and HCl. The alternative microwave method, however, achieves an equivalent Zr recovery of binding gel ZrO_2_ (S1.5. and Table S3). A digestion method with a lower Fe detection limit (0.02 μg Fe cm^–2^, compared to 0.2 μg Fe cm^–2^ for the microwave method) was used for the time- and concentration-dependent Fe-NOM sorption tests. Binding gel discs were digested with *aqua regia* (0.5 mL HNO_3_ and 1.5 mL HCl, 37%) in an open block system (DigiPREP MS, SCP Science) for 2 h at 90 °C and near-dry evaporated at 120 °C. The concentrations of Zr, Ti, and Nb from precipitated metal oxides and of P and Fe from accumulated analytes in the gel digest were measured with ICP-MS (7000, Agilent) (S1.6.). Likewise, the concentrations of these elements were measured in the metal precursor solutions and the initial and final PO_4_, Fe-NOM, and HFO deployment solutions.

### Statistical Analysis and Data Treatment

Statistical analysis was done with JMP (Pro 17, SAS) with comparison of means by pairwise Student’s *t* tests. The content of precipitated metal in the binding gels was always attributed to the whole gel disc (*r* = 1.2 cm). The accumulated analyte in a gel disc was attributed to the contact area with the deployment solution or suspension, disregarding the gel disc’s edge underneath the cap when using a DGT housing (*r* = 1 cm). A binding gel thickness (*x*) of 0.04 cm was assumed in calculations of elemental concentrations expressed per gel volume (M) instead of surface area (μg cm^–2^). All results of P and Fe accumulated in the binding gels reflect the combination of adsorbed analytes and of nonadsorbed analytes, for which no correction was made based on bulk phase concentrations. Errors in this paper consistently refer to standard deviations of replicates (*n* = 2) of analytical procedures.

## Results and Discussion

### Binding Gel Synthesis

#### *In Situ* Precipitated ZrO_2_, TiO_2_, and Nb_2_O_5_ Gels

Increasing the *in situ* precipitated ZrO_2_ concentration in APA gels above the reference 0.1 M Zr was tested for enhancing the binding gel sorption capacity. Binding gel synthesis was achieved up to at least 1 M Zr due to the high water solubility of the conventional ZrOCl_2_ precursor (2 M). A similar concentration increase to 0.3 M Zr was previously implemented for labile silicate measurements.^40^ Here, the binding gel Zr concentration increased linearly with increasing concentration of ZrOCl_2_ in the precursor solution during hydrogel equilibration (Figure 1a). Larger gel than solution concentrations might be attributed to Zr being precipitated on the gel’s exterior from adhering ZrOCl_2_ solution or concentrated in the gel upon matrix shrinkage during its immersion in the buffer. A substantial increase in Zr concentration negatively impacted mechanical and physical hydrogel properties, which could impair analyte diffusion in the binding gel and imaging resolution. These modifications included a diminished gel flexibility and an increased adsorbent distribution heterogeneity (Figure S3).

**Figure 1:**
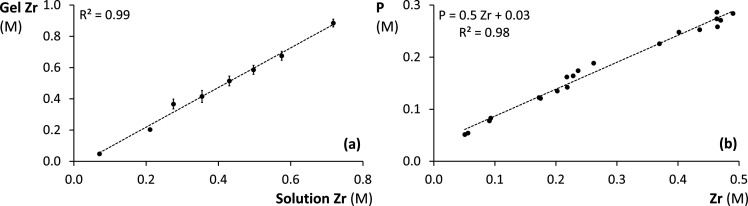
(a) Linear increase of the *in situ* precipitated ZrO_2_ concentration (M Zr) in APA gels with increasing ZrOCl_2_ concentration (M Zr) in the precursor solution. Error bars represent standard deviations (*n* = 2). (b) Linear increase of the total PO_4_ sorption capacity, expressed as the accumulated PO_4_ concentration (M P), with increasing Zr concentration (M) in ZrOCl_2_-based binding gels deployed for 24 h in PO_4_ solutions (400 mg P L^–1^). A linear calibration curve is fitted through the data points of the total PO_4_ sorption capacity.

Decreasing the ZrO_2_ particle size to enhance the binding gel sorption capacity was attempted by using ZrCl_4_ and Zr­(OC_4_H_9_)_4_ instead of ZrOCl_2_. Additionally, novel binding gels of *in situ* precipitated TiO_2_ and Nb_2_O_5_ were speculated to have high NOM affinities. Synthesizing on-target binding gels at 0.1 and 0.3 M Zr, Ti or Nb was not feasible with each metal precursor, despite considerable effort. However, binding gels of two distinct metal concentrations were synthesized from each precursor (Table S4 and Figure S4), all within the range of the ZrOCl_2_-based gels for which PO_4_ sorption was tested (0.05–0.5 M). The alternative precursors reacted vigorously during hydrogel equilibration and *in situ* metal oxide precipitation. The release of a 2-fold more protons by ZrCl_4_ compared to ZrOCl_2_ turned the hydrolysis solutions very acid, but this did not notably affect APA gel integrity. The hydrogels immersed in the poorly soluble Zr­(OC_4_H_9_)_4_ solutions shrank considerably. Precipitation also occurred during hydrogel equilibration in TiCl_4_ solutions, and gel sheets were prone to shrinkage at nominal 0.3 M TiCl_4_ and Ti­(OC_4_H_9_)_4_ concentrations. The dissolution of NbCl_5_ and Nb­(OC_4_H_9_)_5_ in acid solutions slowed down precipitation, although this could not be avoided and affected hydrogel flexibility. Heterogeneities within one binding gel sheet and between identically prepared sheets are introduced when metal oxides precipitate nonuniformly or precursor concentrations are difficult to control. In contrast, a reproducible protocol is needed for homogeneous metal oxide binding gel synthesis for high-resolution imaging applications. Nonetheless, *in situ* TiO_2_ precipitation presumably leads to more homogeneously distributed and small-sized particles in binding gels than Metsorb incorporation. This is desirable for high-resolution imaging and of interest for oxyanions or strongly hydrolyzed species of Al, As, Mo, P, Sb, Se, U, V, and W for which Metsorb-based DGTs are validated^21,28,41–43^ and commercially available (DGT Research Ltd.).

#### Hybrid PVP Gels

Hydrogel matrix adaptation was another strategy to enhance NOM affinity. Semi-interpenetrating APA-PVP gels were synthesized by incorporating up to at least 6% PVP, although hydrogel mechanical strength was increasingly affected. Gel brittleness and phase separation, indicated by a color change from transparent to opaque, intensified with increasing PVP content (Figure S5). These are caused by the diminishing of the APA network cross-link density due to hydrogen bonding between the pyrrolidone group of PVP and the amide bond of polyacrylamide.^34^ Nonetheless, these effects were limited at 1.5% PVP integration in APA or agarose gels, and *in situ* ZrO_2_ precipitation was not hampered (Table S5).

### Sorption Tests in Synthetic Solutions

#### Total PO_4_ Sorption Capacity

The total sorption capacity of the environmentally relevant PO_4_ oxyanion increased linearly with increasing ZrO_2_ concentration up to 0.5 M Zr in ZrOCl_2_-based binding gels (Figure 1b). The maximal PO_4_ accumulation (347 μg P cm^–2^ in a 0.5 M Zr gel) was significantly larger than the uptake by the commercially available 0.1 M Zr gel (83 μg P cm^–2^), for which a DGT capacity of 65 μg P cm^–2^ has been reported^23^ (Figure S6). The calibration curve for PO_4_ accumulation as a function of gel Zr concentration has a slope of 0.5 mol P mol Zr^–1^ and an intercept close to the molar PO_4_ concentration of the deployment solutions (0.014 M). The constant PO_4_ sorption efficiency, represented by the slope, suggests that the ZrO_2_ particle size was independent of the Zr concentration. Additionally, the total PO_4_ capacity was not markedly affected by diffusion impairments due to the hydrogel modifications caused by elevated ZrO_2_ concentrations up to 0.5 M Zr.

The total PO_4_ sorption capacity in gels of alternative metal precursors was large (67–497 μg P cm^–2^), and also increased with increasing gel metal concentration (Figure S6). This concentration dependency prevents direct comparison between different binding gels. However, the molar total PO_4_ sorption capacity of a reference ZrOCl_2_-based gel of equal metal content was predicted for each gel type with the PO_4_ accumulation calibration curve of ZrOCl_2_-based gels (Figure 1b). Consequently, a gel’s molar total PO_4_ sorption capacity was expressed relative to the one predicted for its reference ZrOCl_2_-based gel. This ratio is termed the relative total PO_4_ accumulation (Figure 2). The Zr­(OC_4_H_9_)_4_-based gels were in absolute values (μg P cm^–2^) and relative to their metal concentration (mol P mol metal^–1^) the most enriched in PO_4_, thus yielding the highest relative total PO_4_ accumulations (>1). However, the gel discs substantially expanded during deployment of the Zr­(OC_4_H_9_)_4_-based gels that had previously shrunk during *in situ* precipitation. The additional gel volume was assumed to have a concentration of nonbound PO_4_ equal to that of the bulk solution (400 mg P L^–1^). Subtraction of this supplementary PO_4_ indicated a similar PO_4_ adsorption as in ZrOCl_2_-based gels (details not shown). In contrast, the ratio was <1 in the gels of other metal precursors, i.e., their PO_4_ accumulation was chiefly lower than that of an equivalent ZrOCl_2_-based gel. Overall, ZrO_2_ gels accumulated significantly (*p* < 0.01) more PO_4_ than TiO_2_ and Nb_2_O_5_ gels. The difference in absolute PO_4_ uptake between Ti- or Nb-based gels and gels of Zr precursors increased with increasing metal concentration, except for NbCl_5_ (Figure S8). Lastly, the PO_4_ accumulation did not significantly differ between gels of chloride and n-butoxide metal precursors.

**Figure 2:**
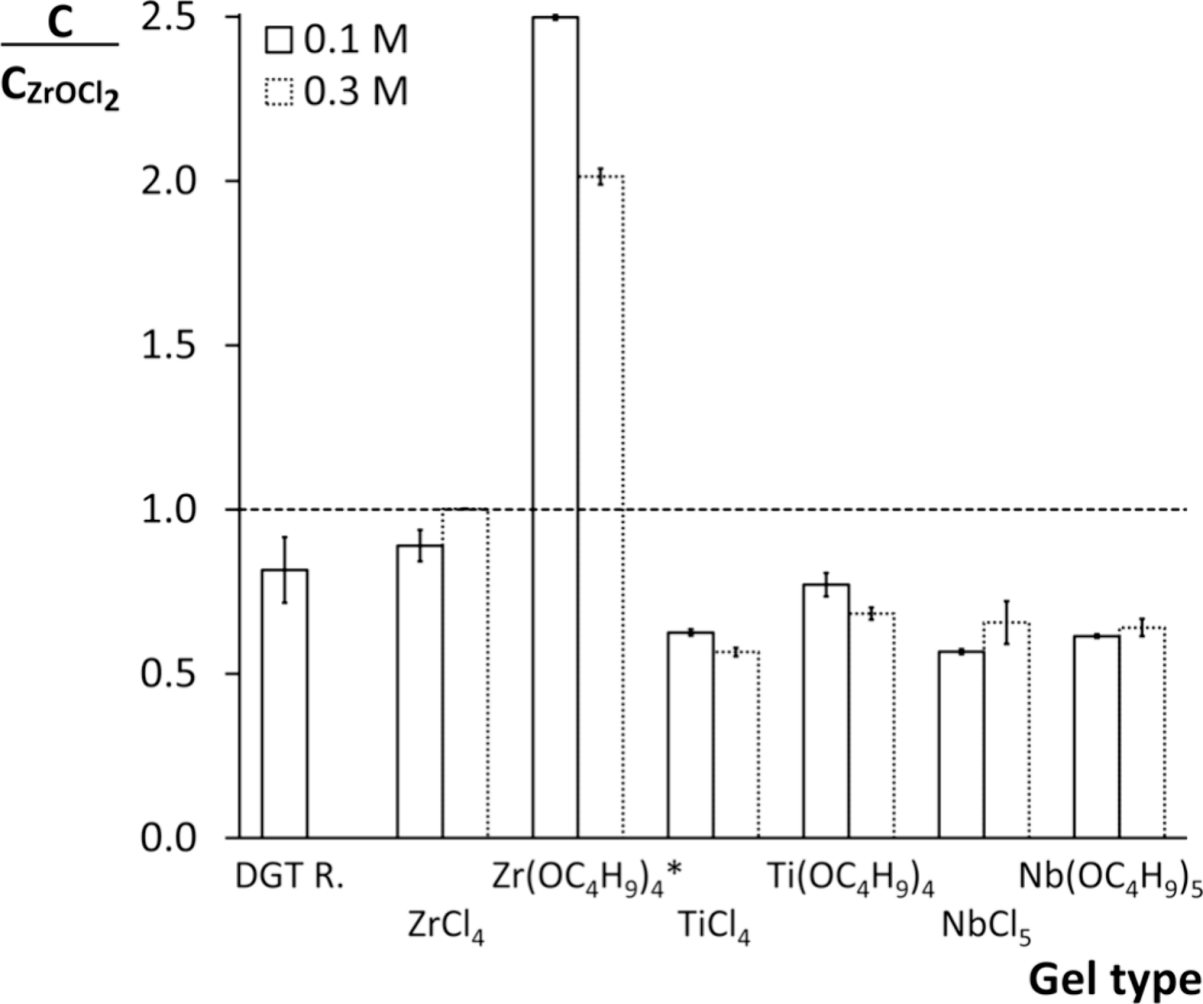
Relative total PO_4_ accumulation of binding gels of different precursors with nominal 0.1 and 0.3 M metal concentrations. This ratio is expressed as a gel’s total PO_4_ sorption capacity (*C*) as a fraction of a ZrOCl_2_-based gel’s capacity at equal molar metal concentration (*C*_ZrOCl2_). The dashed line indicates a relative total PO_4_ accumulation of 1, and error bars represent standard deviations (*n* = 2). *Zr­(OC_4_H_9_)_4_-based gels expanded during deployment, resulting in additional PO_4_ uptake in the gel matrix and an apparent higher (relative) PO_4_ accumulation (see result).

#### Sorption Affinity of Fe-NOM Colloids

The potential of ZrO_2_ binding gels to accumulate small Fe-NOM colloids was examined for ZrOCl_2_-based gels with metal concentrations (0.05–0.4 M) that covered the range of the nominal 0.1 M metal gels of all other precursors (Figure 3). The Fe-NOM colloids were concentrated as Fe concentrations (M) in the gels were 10–50 times higher than in the final deployment suspension, which confirms the anticipated binding to the metal oxides. The accumulation of Fe-NOM did not increase linearly with increasing Zr concentration over the whole concentration range, in contrast with that of PO_4_. The Fe-NOM content increased to a maximum of 6.1 μg Fe cm^–2^ in a 0.18 M Zr gel, beyond which it declined with increasing Zr concentration. Meanwhile, the Fe-NOM sorption efficiency (mmol Fe mol Zr^–1^) of ZrOCl_2_-based gels decreased linearly with increasing Zr concentration of 0.05–0.24 M and was the lowest for the 0.4 M Zr gel (Figure S7). Additionally, heterogeneities in the 0.24 and 0.4 M Zr gels could be observed by nonevenly colored patches of NOM enrichment (Figure S8). This suggests more pronounced impairment of colloid permeation in binding gels with increased metal oxide concentrations due to smaller pore sizes, which was also observed during time-dependent Fe-NOM sorption (see last section). Furthermore, colloids might block hydrogel pores upon adsorption and impede the permeation and accumulation of subsequently arriving analytes. The inherently lower diffusion coefficient of Fe-NOM (1.65 × 10^–6^ cm^2^ s^–1^ in water, calculated with the Stokes–Einstein equation) than PO_4_ (5.27 × 10^–6^ cm^2^ s^–1^ in APA gel, provided by DGT Research Ltd.) also makes these colloids more prone to diffusion impairment. The maximum Fe-NOM content in 0.18 M Zr gels corresponds to a total OC content of 1.6 mol OC mol Zr^–1^. A fraction of 0.16 mol OC mol Zr^–1^ is assumed to be directly bound to ZrO_2_, considering a negative charge of 0.1 mol mol OC^–1^ at pH 6 for SRNOM due to carboxylate and phenolate functional groups.^44^ This is lower than the PO_4_ sorption efficiency of 0.5 mol P mol Zr^–1^ in ZrOCl_2_-based gels because of the previously discussed declining Fe-NOM sorption efficiency with increasing gel Zr concentration.

**Figure 3:**
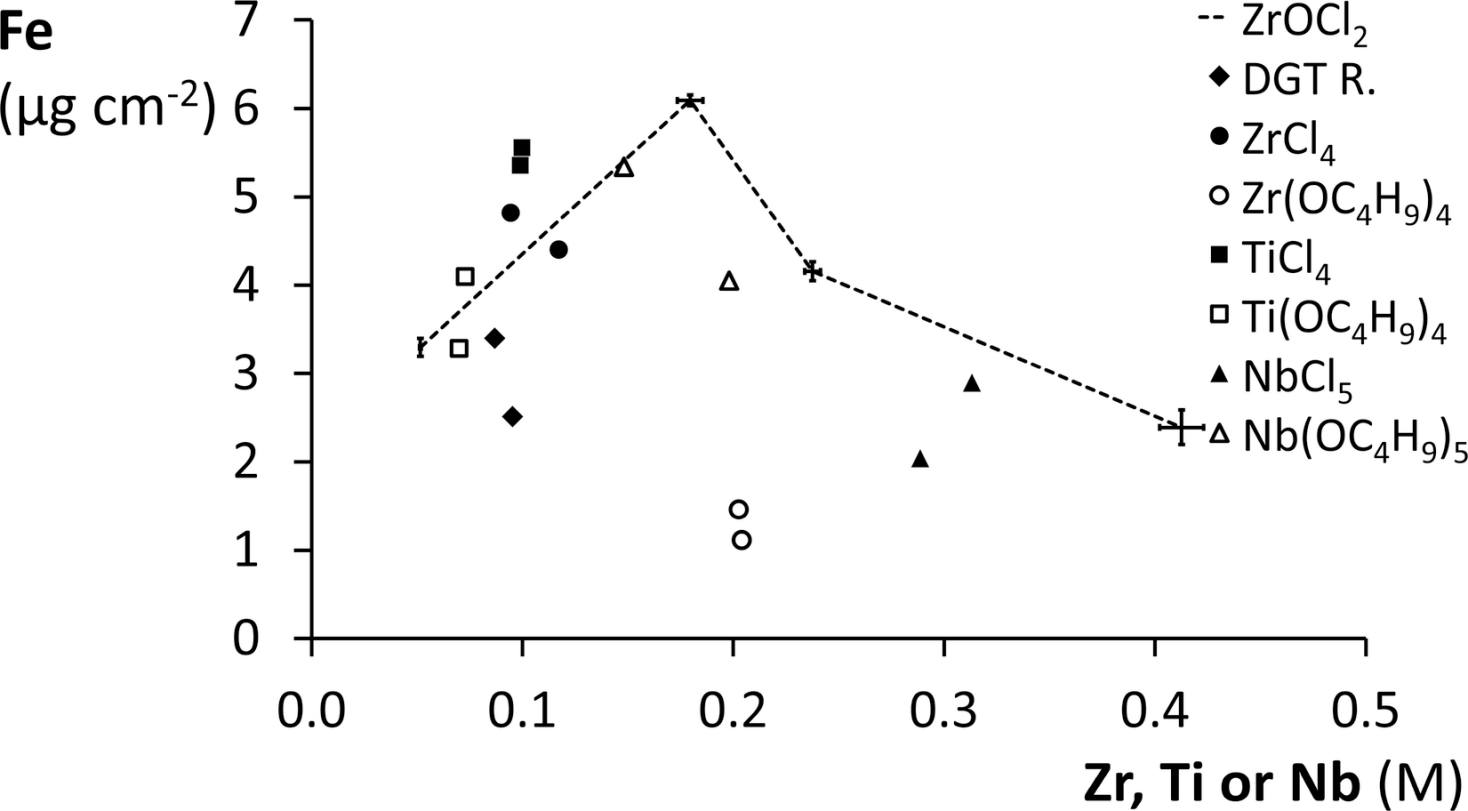
Accumulated Fe-NOM colloids (μg Fe cm^–2^) in binding gels of different metal precursors as a function of their measured metal concentration (M). The gels were simultaneously deployed in DGT housings in a near-constant Fe-NOM suspension (5 mg Fe L^–1^) for 24 h. The data points of ZrOCl_2_-based reference gels are connected to guide the eye, and their error bars represent standard deviations (*n* = 2).

Gels of alternative metal precursors followed this trend of decreasing Fe-NOM sorption efficiency with increasing metal concentration (Figures 3 and S7), presumably linked to impaired Fe-NOM permeation. Large replicate standard deviations indicate metal oxide heterogeneities within binding gel sheets of some precursors. The Zr­(OC_4_H_9_)_4_-based gel, which did not expand during deployment in a DGT housing, had the lowest Fe-NOM uptake and sorption efficiency despite its intermediate metal concentration. This also contrasted with its high total PO_4_ sorption capacity, even when considering the gel expansion during PO_4_ sorption. Colloid diffusion impairment in the severely shrunken and rigid gel supposedly caused low Fe-NOM accumulation. The commercially available ZrO_2_ gel had the lowest Fe-NOM uptake and sorption efficiency among gels of similar metal concentration. Binding gels of TiO_2_ and Nb_2_O_5_ were hypothesized to have a larger NOM affinity than ZrO_2_ gels due to the higher electronegativities of Ti and Nb than Zr. Nevertheless, their Fe-NOM contents, like their total PO_4_ sorption capacities, were generally lower than those of ZrOCl_2_-based gels. Only the TiCl_4_-based gel had a Fe-NOM accumulation and sorption efficiency higher than those of equivalent ZrOCl_2_-based gels. This still suggests a larger NOM affinity than in other gel types, although its low total PO_4_ sorption capacity is a disadvantage for oxyanion competition (Figure 2). A high Fe-NOM uptake was expected for Nb_2_O_5_ gels as the affinity of large surface area Nb_2_O_5_ particles has been shown to be high for carboxylates, even in the presence of competing species.^29^ Here, the tendency of the Nb precursors to readily precipitate could have promoted larger metal oxide particle sizes, resulting in lower reactive surface areas. The high measured Nb concentrations of the nominal 0.1 M Nb_2_O_5_ gels can explain their low Fe-NOM sorption efficiencies because of the general trend of decreasing Fe-NOM sorption efficiency with increasing metal concentration (Figure S7).

#### Effect of Hydrogel Type on Fe-NOM and HFO Colloid Sorption

The ability to accumulate small Fe-NOM and larger-sized HFO colloids was tested for various hydrogel types with and without ZrO_2_ adsorbent (Figure 4). The Fe-NOM and HFO colloids were, unlike PO_4_, concentrated in ZrO_2_-free APA gels, with factors of 8 and 10 compared to their deployment solutions. It has been shown before that humic acids can accumulate in APA gels by a factor of 10, presumably due to hydrophobic interaction with the gel matrix.^45^ Agarose gels were not enriched in Fe-NOM but accumulated larger HFO particles. The inherent negative charge in agarose gels could rather limit uptake of the more negatively charged Fe-NOM than HFO, whereas the reverse might be true for positively charged APA gels.^46^

**Figure 4:**
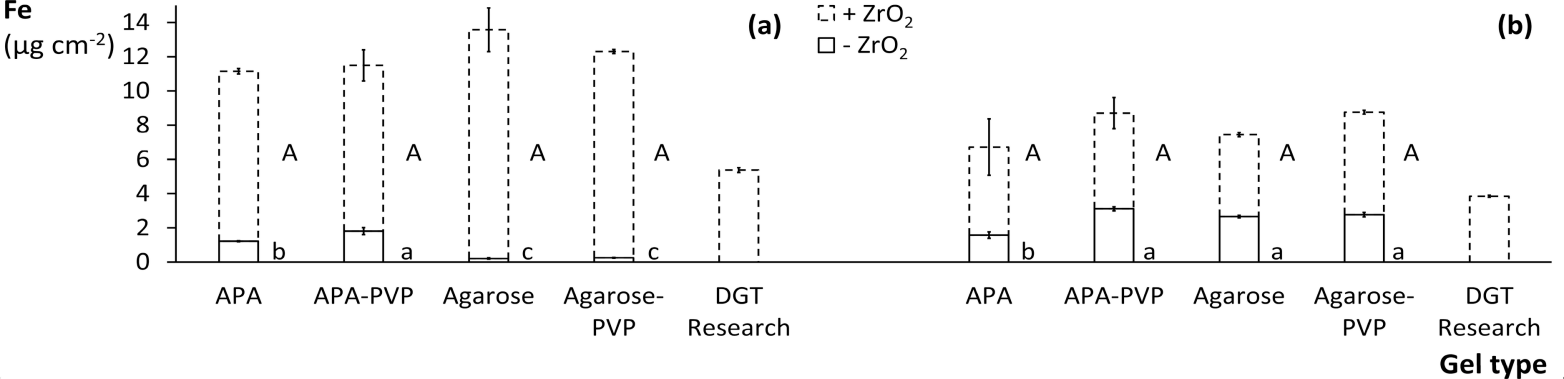
Accumulated (a) Fe-NOM and (b) HFO colloids (μg Fe cm^–2^) in binding gels of various hydrogel types with (+) and without (−) ZrO_2_. The gels were simultaneously deployed in DGT housings in near-constant Fe-NOM or HFO suspensions (5 mg Fe L^–1^) for 24 h. The error bars represent standard deviations (*n* = 2), and significantly different mean Fe-NOM and HFO contents of + and – ZrO_2_ gels are denoted with different letters (*p* < 0.05).

The interaction of NOM with both PVP, via hydrogen binding, and ZrO_2_, via ligand exchange, allows these adsorbents to accumulate colloids cooperatively. Incorporating PVP only increased colloid uptake in APA gels to a significant, albeit limited, extent, but it could be a valuable selective NOM adsorbent in the presence of competing PO_4_. The *in situ* precipitated ZrO_2_ greatly increased Fe-NOM adsorption, by 10 (APA) and 13 μg Fe cm^–2^ (agarose), while HFO uptake increased by 5 μg Fe cm^–2^. The Zr concentrations in APA- and agarose-based gels differed by a factor of 1.5, although they were simultaneously immersed in a single ZrOCl_2_ and buffer solution (Table S5). The absolute Fe-NOM and HFO contents of APA­(-PVP) gels were, despite higher Zr concentrations, not significantly different from those of agarose­(-PVP) gels. The hypothesized benefit of larger pore sizes in agarose gels could not be ascertained because the inherent sorption properties and the precipitated ZrO_2_ concentrations of the APA and agarose gels differed. Analysis of colloid permeation and partitioning in the hydrogel pores and of the main binding mechanisms might elucidate this. All gels, however, significantly differed from the commercially available gel that adsorbed the least of both Fe-NOM and HFO, either in absolute values or normalized per unit Zr concentration. Summarizing, hydrogels with PVP and/or agarose are not preferred over APA gels because their colloid accumulation is not distinctly superior, whereas their fragility complicates imaging applications.

#### Accumulation of Colloidal Fe

The Fe uptake by ZrOCl_2_-based binding gels deployed with and without a colloid-excluding dialysis membrane in Fe-NOM and HFO suspensions was compared to verify that colloidal Fe can be detected on binding gels. Although the dialysis membranes did not prohibit Fe (Figure 5) and NOM (Figure S9) uptake by the binding layers, the binding gels deployed without a dialysis membrane had much higher Fe contents, which indicates that colloidal Fe is the major source of Fe accumulated on the binding gels.

**Figure 5:**
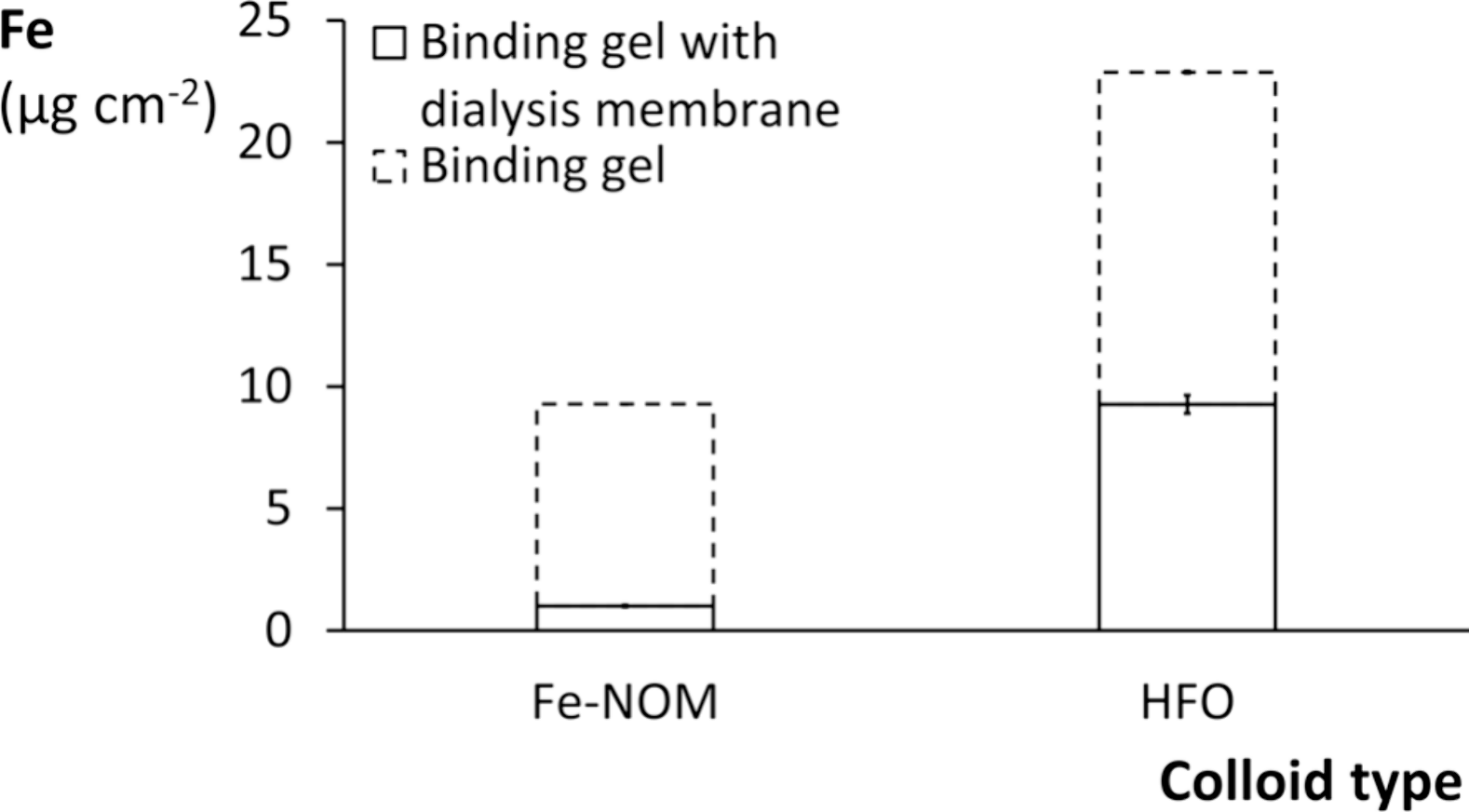
Accumulated Fe (μg cm^–2^) in ZrOCl_2_-based binding gels (0.2 M Zr) with and without colloid exclusion by a dialysis membrane. The gels were simultaneously deployed in DGT housings in near-constant Fe-NOM and HFO suspensions (5 mg Fe L^–1^) for 24 h. The error bars represent standard deviations (*n* = 2).

#### Time- and Concentration-Dependent Fe-NOM Sorption

A 0.2 M ZrOCl_2_-based binding gel was put forward for further testing because of its superior sorption properties relative to the 0.1 M Zr reference. The 0.1 and 0.2 M Zr gels were subjected to time- and concentration-dependent Fe-NOM sorption (Figure 6). Both binding gels initially acted as a zero sink because the Fe-NOM uptake linearly increased with increasing deployment times at an equal rate of 0.18 ng Fe cm^–2^ s^–1^ (Figures 6a and S10a). The Fe-NOM accumulation by the two gel types diverged with a decrease in uptake rate after 10 min for the 0.2 M Zr gel and after 1 h for the 0.1 M Zr gel. After 24 h, the Fe-NOM accumulation did not yet reach an equilibrium and was still larger in the 0.1 M than in the 0.2 M Zr gels. Higher accumulation is expected at longer deployment times due to slow Fe-NOM permeation into the gels, which is likely more pronounced in gels of increased Zr concentration. However, uptake rates during DGT deployment are rather determined by the supply rate from the bulk phase than by diffusion and sorption in the binding gels.

**Figure 6:**
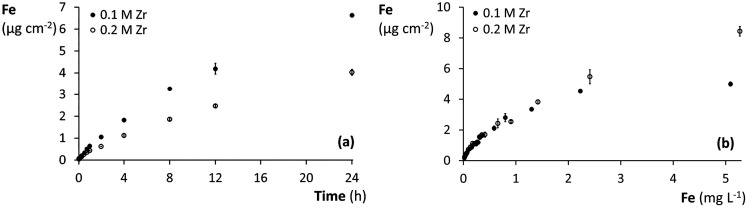
Uptake of Fe-NOM colloids (μg Fe cm^–2^) in 0.1 and 0.2 M ZrOCl_2_-based binding gels as a function of (a) time and (b) final Fe-NOM concentration in suspension. The gel discs were (a) simultaneously deployed in a Fe-NOM suspension (0.5 mg Fe L^–1^) between 1 min and 24 h, and (b) individually deployed in DGT housings in Fe-NOM suspensions with 0.05–5 mg Fe L^–1^ initial concentrations for 24 h. The error bars represent standard deviations (*n* = 2).

The Fe-NOM accumulation by 0.1 and 0.2 M ZrOCl_2_-based binding gels also linearly increased with increasing Fe-NOM concentration in suspension (Figures 6b and S10b). The Fe-NOM uptake was consistent for both gel types up to 1.5 mg Fe L^–1^ in suspension, with a linear increase between 0.02 and 0.17 mg Fe L^–1^. The Fe-NOM uptake in the 0.1 M Zr gels stagnated at a total Fe-NOM sorption capacity of 5 μg Fe cm^–2^. Meanwhile, the accumulation in the 0.2 M gels further increased with increasing Fe concentration, and the sorption capacity was not yet reached at the final suspension concentration of 5 mg Fe L^–1^. The Fe-NOM uptake of both gel types deployed for 24 h in an initial 0.5 mg Fe L^–1^ suspension was markedly higher in the kinetics test (Figure 6a, 6.6 and 4.0 μg Fe cm^–2^) compared to the concentration test (Figure 6b, 1.6 and 1.7 μg Fe cm^–2^). This is due to differences in their experimental setup. In the first case, multiple gels were freely deployed in a well-mixed suspension with a good gel-suspension contact. The gels in the concentration experiment, however, were individually deployed in DGT housings that restricted diffusion to one direction and could be more prone to diffusive boundary layer formation. In addition, Fe in the kinetics test was depleted by only 12% due to the large suspension volume (equivalent to 75 mL per gel disc) and the low Fe-NOM uptake in gels with short deployment times. During the concentration test, 50 mL of an equivalent 0.5 mg Fe L^–1^ Fe-NOM suspension was depleted by more than 30% by one gel disc.

## Conclusions

The DGT technique is a promising method for *in situ* sampling and high-resolution imaging of natural soil colloids. This study aimed to tackle an important first step by optimizing a binding layer for organomineral Fe colloids. A 0.2 M ZrO_2_ binding gel of *in situ* precipitated ZrOCl_2_ in an APA hydrogel is proposed. It was selected after comparing the accumulation of PO_4_ and organomineral Fe colloids on original binding layers of ZrO_2_, TiO_2_, and Nb_2_O_5_ adsorbents in PVP, APA, and agarose hydrogels. The 0.2 M ZrO_2_ gel is synthesized similarly to the existing *in situ* precipitated 0.1 M ZrO_2_ DGT binding gel, except for the increased ZrOCl_2_ and MES buffer concentrations, and has superior sorption properties. The following steps will assess DGT deployment of the binding layer with a diffusion layer suitable for colloid sampling under environmentally relevant conditions. Validation of the DGT performance is required for diverse natural soil colloids and test conditions, e.g., pH, ionic strength, and solute composition. An in-depth investigation of diffusion-dependent competition effects of anionic species on the DGT in complex systems is also essential. Finally, soil application of the DGT for *in situ* natural soil colloid sampling should be optimized, considering appropriate deployment times, and verified against traditional soil solution sampling methods. Attention should go to differentiating between DGT-accumulated solutes and colloidal species. The desired outcome is to establish the proposed DGT method for *in situ* quantitative analysis and LA-ICP-MS high-resolution imaging of natural colloids in soil.

## Supplementary Material



## Notes

Additional experimental details of binding gel synthesis and analysis, and supplementary results of binding gel synthesis and sorption tests (PDF)
