# Supplementary material for: Binding Gel Optimization for the Detection of Organomineral Iron Colloids with the Diffusive Gradients in Thin Films Method
Source: Anal Chem. 2025 Nov 24;97(48):26384–92. doi: 10.1021/acs.analchem.5c02501 (PMC12874203; doi:10.1021/acs.analchem.5c02501)
Supplement: Supplementary file 1 [file ac5c02501_si_001.pdf]

Supporting Information

Binding Gel Optimization for the Detection of Organomineral Iron Colloids with the Diffusive Gradients in Thin Films Method

Jasnalien Ceulemans,* Claudia Moens, and Erik Smolders

Division of Soil and Water Management, Department of Earth and Environmental Sciences, KU Leuven, Kasteelpark Arenberg 20, 3001 Heverlee, Belgium

Email: jasnalien.ceulemans@kuleuven.be.

TABLE OF CONTENTS

S1. EXPERIMENTAL SECTION S3
S1.1. Hydrogel Synthesis S3
APA Hydrogels S3
Agarose Hydrogels S3
Hybrid PVP Hydrogels S4
S1.2. Metal Precursor Hydrolysis S4
S1.3. Synthetic Fe-NOM and HFO Colloids S5
S1.4. Binding Gel Elution S5
S1.5. Metal Oxide Digestion S6
S1.6. Elemental Analysis S6
S2. RESULTS AND DISCUSSION S7
S2.1. Binding Gel Synthesis S7
In situ Precipitated ZrO_2, TiO_2, *and* Nb_2O_5 *Gels* S7
Hybrid PVP gels S8
S2.2. Sorption Tests in Synthetic Solutions S9
Total PO_4 *Sorption Capacity* S9
Sorption Affinity of Fe-NOM Colloids S9
Accumulation of Colloidal Species S10
Time- and Concentration-Dependent Fe-NOM Sorption S11
S2.3. Summarizing Table S12

S1. EXPERIMENTAL SECTION

Table S1. Overview of all synthesized binding layers of *in situ* precipitated ZrO_2, TiO_2, and Nb_2O_5 in hydrogels with the analytes for which sorption was tested, phosphate (PO_4) and colloids of Fe-NOM complexes (Fe-NOM) and NOM-coated hydrous ferric oxides (HFO).

**Binding layer**			**Sorption test**
Metal precursor	Nominal metal concentration	Hydrogel	Analyte
$ZrOCl_2$	0.1-0.5 M	APA	PO_4; Fe-NOM
$ZrCl_4$	0.1 & 0.3 M	APA	PO_4; Fe-NOM
$Zr(OC_4H_9)_4$	0.1 & 0.3 M	APA	PO_4; Fe-NOM
$TiCl_4$	0.1 & 0.3 M	APA	PO_4; Fe-NOM
$Ti(OC_4H_9)_4$	0.1 & 0.3 M	APA	PO_4; Fe-NOM
$NbCl_5$	0.1 & 0.3 M	APA	PO_4; Fe-NOM
$Nb(OC_4H_9)_5$	0.1 & 0.3 M	APA	PO_4; Fe-NOM
$ZrOCl_2$	0 & 0.1 M	APA	Fe-NOM; HFO
$ZrOCl_2$	0 & 0.1 M	APA-PVP	Fe-NOM; HFO
$ZrOCl_2$	0 & 0.1 M	Agarose	Fe-NOM; HFO
$ZrOCl_2$	0 & 0.1 M	Agarose-PVP	Fe-NOM; HFO

APA: agarose derivative-crosslinked polyacrylamide; PVP: polyvinylpyrrolidone

S1.1. Hydrogel Synthesis

APA Hydrogels

Hydrogels of APA were prepared according to standard procedures.[1,2] A gel solution was prepared with 15% agarose-derived cross-linker (2%, DGT Research Ltd.), 37.5% acrylamide solution (40%) and 47.5% ultrapure (Milli-Q) water. To 5 mL of gel solution, 35 µL of freshly prepared ammonium persulphate solution (APS, 10% m/v) and 12.5 µL of tetramethylethylenediamine (TEMED) were added. Subsequently, this mixture was cast between two acid-washed glass plates (17 cm x 6 cm) separated by a 0.25 mm-thick spacer and allowed to polymerize for 1 h at 45 °C. Gels were hydrated for 24 h to a thickness of 0.4 mm in several times replenished Milli-Q water and afterwards stored in a 10 mM sodium chloride (NaCl) solution.

Agarose Hydrogels

Agarose gels were prepared similarly to Zhang and Davison,[3] by dissolving 2% m/v agarose in Milli-Q water through microwave-assisted heating until the solution turned transparent. The thoroughly mixed gel solution was immediately cast between two preheated, acid-washed glass plates separated by a

0.4 mm-thick spacer, as these gels are not prone to swelling by hydration. After gelation at room temperature for 1 h, the gels were washed in Milli-Q water and stored in a 10 mM NaCl solution. The *in situ* precipitation of ZrO_2 from $ZrOCl_2$ in agarose gels was done as previously described.

Hybrid PVP Hydrogels

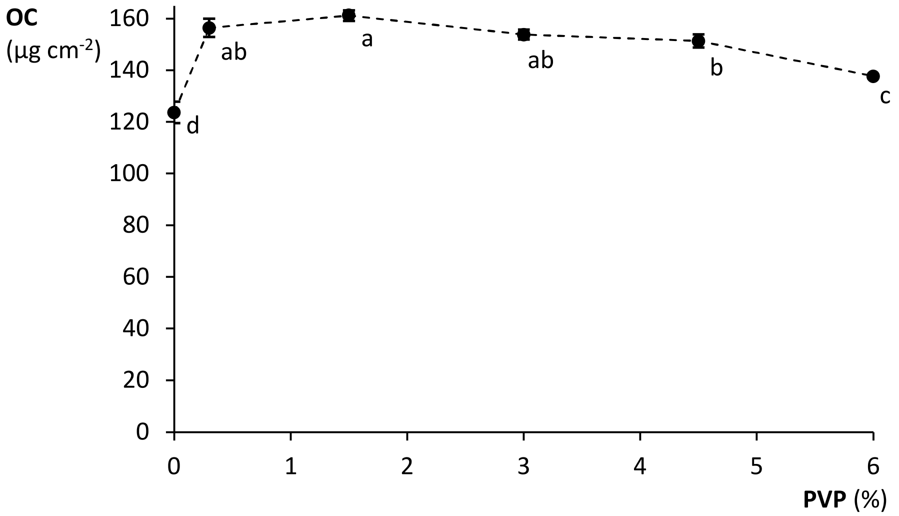

Figure S1. Accumulated SRNOM (µg OC cm^{-2}) in ZrO_2 APA binding gels of different PVP content (%). Gel discs (n = 2, r = 1.2 cm) were individually deployed in a SRNOM suspension (400 mg OC L^{-1}) for 24 h and eluted in 0.2 M NaOH for 24 h with OC measured spectrophotometrically (λ = 254 nm, UV-1900i, Shimadzu). Data points are connected to guide the eye for easy comparison, error bars represent standard deviations (n = 2), and statistically significant mean SRNOM contents are denoted with different letters ($p < 0.05$).

S1.2. Metal Precursor Hydrolysis

The metal precursors react according to the following reaction equations in water and acid solutions:

$ZrOCl_2$

$ZrOCl_2 + 3H_2O \leftrightarrow Zr(OH)_4^{\circ} + 2\ H^+ + 2\ Cl^-$ (Equation S1)

Metal chloride

$ZrCl_4 + 4H_2O \leftrightarrow Zr(OH)_4^{\circ} + 4\ H^+ + 4\ Cl^-$ (Equation S2)

Metal alkoxide

$Zr(OR)_4 + 2H_2O \leftrightarrow ZrO_2 + 4ROH$ (Equation S3)

$Zr(OR)_4 + 4H_3O^+ \leftrightarrow Zr(OH)_4^{\circ} + 4\ H^+ + 4ROH$ (Equation S4)

S1.3. Synthetic Fe-NOM and HFO Colloids

Synthetic organomineral Fe colloids were previously characterized by Moens *et al*. with various new and standard techniques: Flow Field Flow Fractionation coupled with an UV/VIS detector and ICP-MS (FlFFF-UV-ICP-MS), single-particle ICP-MS, dynamic light scattering (DLS), nanoparticle tracking analysis (NTA), transmission electron microscopy (TEM), membrane filtration, centrifugation and dialysis.[4] Here, the preparation of the freshly synthesized Fe-NOM and HFO colloids (previously reported as A-110 and A-2)[5] was verified with FlFFF-UV-ICP-MS (AF2000, Postnova Analytics; SPD-20A, Postnova Analytics; 7000, Agilent). Similar signals were obtained for UV (data not shown) and Fe detected with ICP-MS (Figure S2) as a function of elution time, which reflect the size distribution of the organomineral Fe colloids.[5]

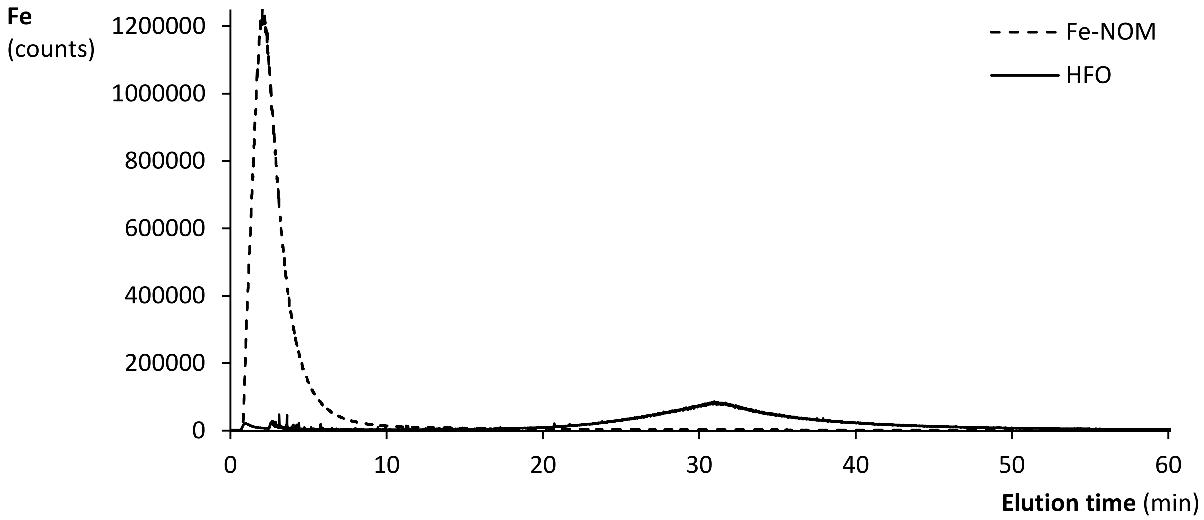

Figure S2: Signal (counts) of Fe as a function of elution time of freshly synthesized Fe-NOM and HFO colloids (which reflect their size distribution),[5] obtained with FlFFF-ICP-MS.

S1.4. Binding Gel Elution

To determine the elution efficiency, discs (n = 4, r = 1.2 cm) of a 0.1 M $ZrOCl_2$-based binding gel were deployed in 50 mL of both undiluted and ten times diluted Fe-NOM suspension (0.5 and 5 mg Fe L^{-1} in 10 mL NaCl at pH 6) for 24 h at 20 °C on the end-over-end shaker. Two replicates were eluted in 2 mL of 0.2 M NaOH for 24 h, whereas the other two were digested with *aqua regia* in a block digestion system.

The concentration of Fe in the digests and the initial and final deployment solutions was measured with ICP-MS. The OC content of eluates and deployment solutions was determined by non-purgeable OC measurement (TOC-L, Shimadzu).

Table S2. Accumulation of Fe-NOM in 0.1 M $ZrOCl_2$-based Zr APA gels deployed in suspensions of low and high Fe-NOM concentration determined by digestion and elution. The efficiency of Fe-NOM digestion and elution is expressed as the ratio of Fe or OC in digested and eluted gels over the gel Fe or OC content calculated as the difference between initial and final Fe concentrations. Standard deviations are denoted (n = 2).

**Deployment suspension**	**Suspension** (mg Fe L^{-1})		**Gel** (μg Fe cm^{-2})		**Efficiency** (%)	**Suspension** (mg OC L^{-1})		**Gel** (μg OC cm^{-2})		**Efficiency** (%)
	Initial	Final	Calculated	Digested	Digestion	Initial	Final	Calculated	Eluted	Elution
Fe-NOM_{low}	0.4	0.2 ± <0.1	2.0 ± 0.4	2.1 ± 0.5	108 ± 2	9.5	5.0 ± 0.3	49 ± 3	41 ± 4	84 ± 3
Fe-NOM_{high}	4.7	3.6 ± 0.1	12 ± 2	11 ± 2	90 ± 5	95	86 ± 1	100 ± 6	98 ± 4	98 ± 10

S1.5. Metal Oxide Digestion

In each gel digestion, blank samples and the certified reference materials (CRMs) NIM-L (Lujavrite, SARM 3, SA Bureau of Standards) and OREAS 47 (Anomalous Glacial Till, ORE) as internal reference were included.

Table S3. Absolute and relative (ratio) recovery of Zr by digestion with the 4-acids hot plate and the HNO_3-H_2SO_4 microwave method of a commercially available ZrO_2 (DGT Research) and a $ZrOCl_2$-based binding gel, and of Oreas 47 and NIM-L Lujavrite CRMs. The Zr concentration was measured in the digests with ICP-MS and presented with its standard deviation (n = 2). The Zr recovery of the binding gels was similar between the two methods (ratio = 1), whereas lower Zr contents were obtained by the microwave method for rather recalcitrant ZrO_2 in the CRMs.

**Sample**	**4-acids method** (μg Zr cm^{-2} or μg Zr g^{-1})	**HNO_3-H_2SO_4 method** (μg Zr cm^{-2} or μg Zr g^{-1})	**Ratio** (-)
DGT Research gel	275 ± 20	296 ± 19	1.07
$ZrOCl_2$ gel	87 ± 10	86 ± 2	0.99
Oreas 47	79 ± 3	40 ± 3	0.51
NIM-L Lujavrite	10026 ± 41	6439 ± 3	0.64

S1.6. Elemental Analysis

Samples were measured with ICP-MS by the direct injection method, in which the uptake circuit between sample and nebulizer was shortened to diminish adherence of Zr, Ti and Nb to tubing and consequential memory effects. Rhodium (Rh) was spiked to all samples as an internal standard for the correction of

matrix effects and instrumental drift. In each batch the CRMs TM (TM-27.4 Environmental Matrix Reference Material, Canada), SPS (SPS-SW2 Batch 140 Reference Material for Measurement of Elements in Surface Waters, Spectrapure Standards) and NIST (SRM 1643f Trace Elements in Water, National Institute of Standards and Technology) were analyzed regularly to verify the accuracy of the measurement (10% deviation from certified concentrations allowed). The ICP-MS used the *No gas mode* for measurements of Zr, Ti and Nb, the *He mode* for Fe and the *High Energy He mode* for P.

S2. RESULTS AND DISCUSSION

S2.1. Binding Gel Synthesis

In situ Precipitated ZrO_2, TiO_2, and Nb_2O_5 Gels

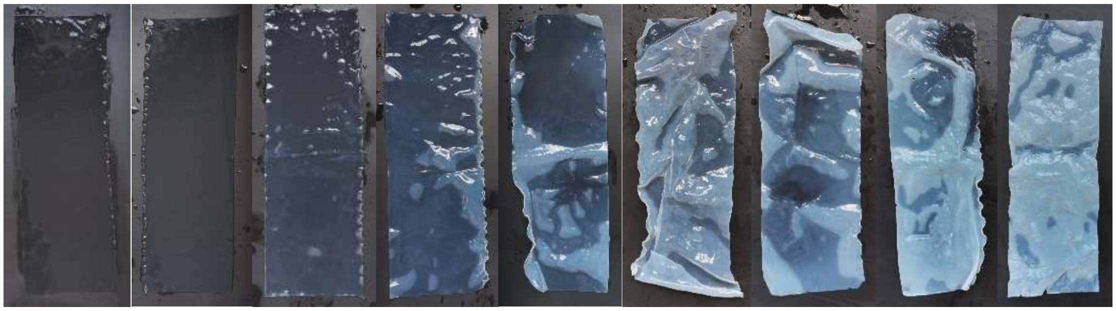

Figure S3. Gel sheets of $ZrOCl_2$-based binding gels of increasing Zr concentration (0.1-0.9 M Zr, left to right). Gel rigidity and heterogeneity increased with increasing Zr content.

Table S4. Nominal (theoretical) metal precursor concentrations in the synthesis solutions versus measured concentrations of Zr, Ti, and Nb (M) precipitated as metal oxides in APA gels with their standard deviations (n = 2). The measured concentrations (µg cm^{-2}) were converted to molar concentrations (M) based on the gel disc dimensions (x = 0.04 cm) to facilitate comparison with their respective nominal concentrations.

	**Nominal metal concentration**	
	0.1 M	0.3 M
**Metal precursor**	**Measured gel Zr, Ti or Nb concentration** (M)	
$ZrCl_4$	0.14 ± <0.01	0.26 ± <0.01
$Zr(OC_4H_9)_4$	0.19 ± 0.01	0.29 ± <0.01
$TiCl_4$	0.10 ± <0.01	0.36 ± <0.01
$Ti(OC_4H_9)_4$	0.08 ± <0.01	0.39 ± <0.01
$NbCl_5$	0.26 ± 0.02	0.30 ± 0.02
$Nb(OC_4H_9)_5$	0.17 ± <0.01	0.27 ± 0.02

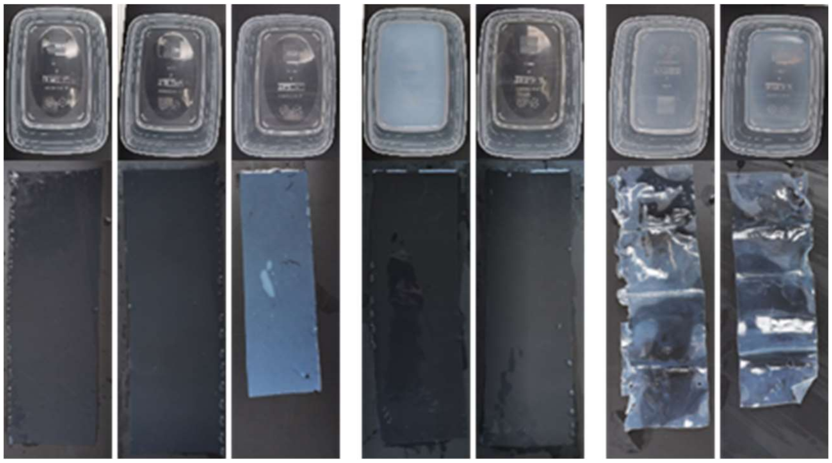

Figure S4. Metal solutions and binding gel sheets of the nominal 0.1 M metal precursors, $ZrOCl_2$, $ZrCl_4$, $Zr(OC_4H_9)_4$, $TiCl_4$, $Ti(OC_4H_9)_4$, $NbCl_5$, and $Nb(OC_4H_9)_5$ (left to right).

Hybrid PVP gels

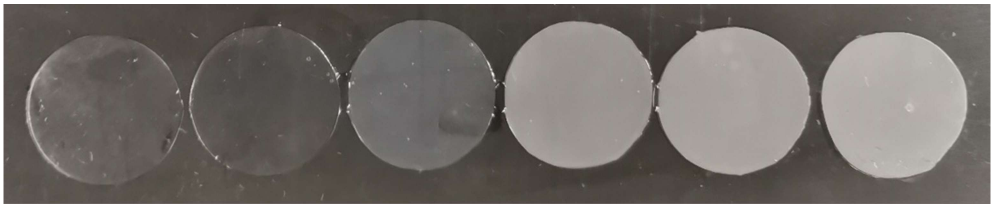

Figure S5. Color change from transparent to opaque due to phase separation in gels of increasing PVP content (0%-6% m/v, left to right).

Table S5. Concentrations of Zr (M) in a commercially available ZrO_2 binding gel (DGT Research) and in gels of different hydrogel types. The Fe-NOM and HFO sorption efficiencies (mmol Fe mol Zr^{-1}) are expressed as the ratio of accumulated Fe over the Zr content of the gels, during deployment in DGT housings in a Fe-NOM or HFO (5 mg Fe L^{-1}) suspension for 24 h. The standard deviations are presented (n = 2).

Gel type	Gel Zr (M)			Sorption efficiency Fe-NOM (mmol Fe mol Zr^{-1})			Sorption efficiency HFO (mmol Fe mol Zr^{-1})		
DGT Research	0.08	±	<0.01	30.7	±	0.2	18.4	±	<0.1
APA	0.14	±	<0.01	38.1	±	2.5	22.1	±	5.6
APA-PVP	0.15	±	<0.01	35.8	±	2.5	26.3	±	2.0
Agarose	0.09	±	<0.01	69.5	±	4.3 *	35.8	±	0.8 *
Agarose-PVP	0.09	±	<0.01	63.2	±	0.3 *	41.3	±	0.5 *

* Significantly different from control (DGT Research) according to Dunnett comparison of means ($p < 0.05$).

S2.2. Sorption Tests in Synthetic Solutions

Total PO4 Sorption Capacity

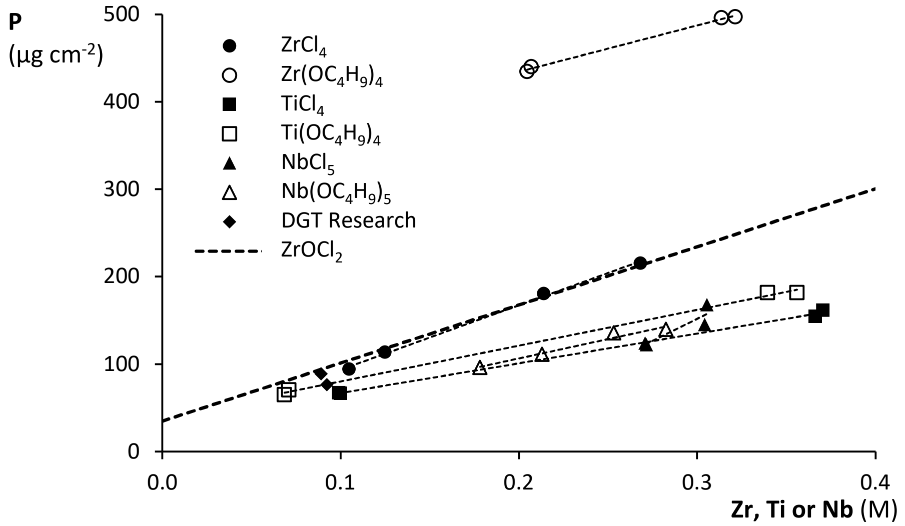

Figure S6. Accumulated PO_4 (µg P cm^{-2}) in binding gels of different metal precursors as a function of their metal concentration (M) with the calibration curve of the total PO_4 sorption capacity of $ZrOCl_2$-based gels. Gel discs (n = 2) were deployed for 24 h in PO_4 solutions (400 mg P L^{-1}).

Sorption Affinity of Fe-NOM Colloids

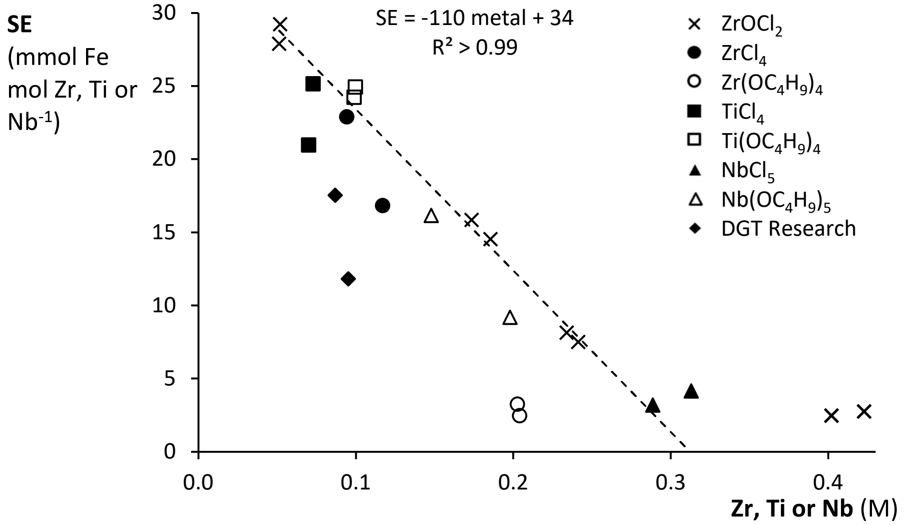

Figure S7. Sorption efficiency of Fe-NOM colloids (SE, mmol Fe mol $metal^{-1}$) of binding gels of different metal precursors, expressed as the accumulated Fe-NOM relative to the metal content, as a function of their metal concentration (M). Gel discs were simultaneously deployed in DGT housings in a near-constant Fe-NOM suspension (5 mg Fe L^{-1}) for 24 h. A linear trendline was fitted through the data points of 0.05-0.24 M $ZrOCl_2$-based reference gels. Error bars represent standard deviations (n = 2).

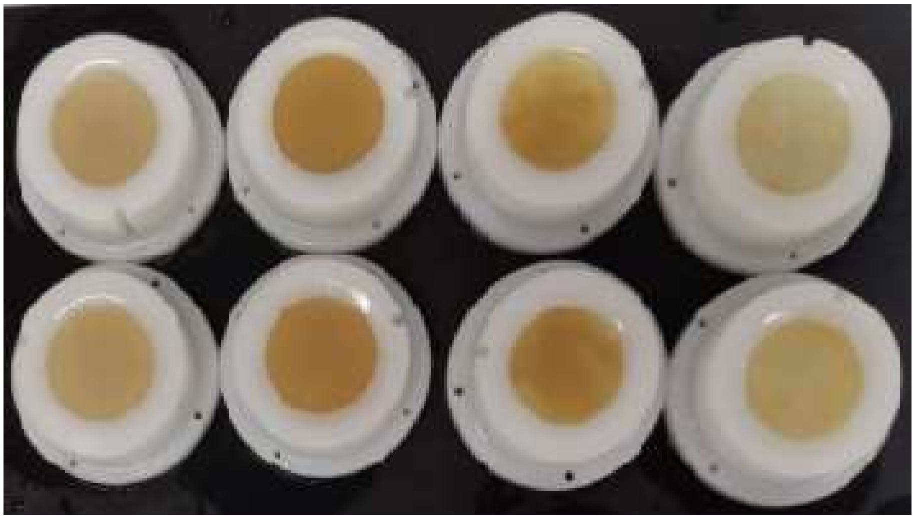

Figure S8. Coloration due to Fe-NOM accumulation (Figure 3) in 0.05-0.4 M $ZrOCl_2$-based gels. The gel Zr concentration increases (0.05-0.24 M, left to right), and replicates (n = 2) are positioned in the vertical direction. Heterogeneities in bound Fe-NOM are visible in the gels with the highest Zr concentrations.

Accumulation of Colloidal Species

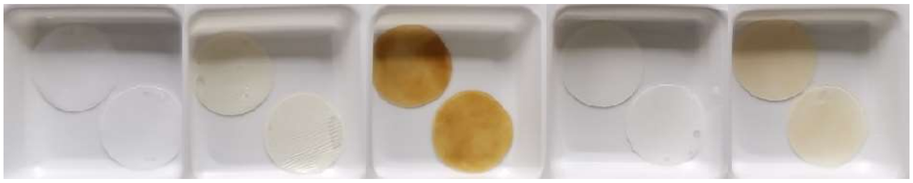

Figure S9. Coloration due to NOM uptake in 0.2 M $ZrOCl_2$-based gels (n = 2). The binding gels were deployed for 24 h in a 10 mM NaCl solution (blank), in a Fe-NOM suspension (5 mg Fe L^{-1}) with and without colloid exclusion by a dialysis membrane, and in a HFO suspension (5 mg Fe L^{-1}) with and without colloid exclusion by a dialysis membrane (left to right).

Time- and Concentration-Dependent Fe-NOM Sorption

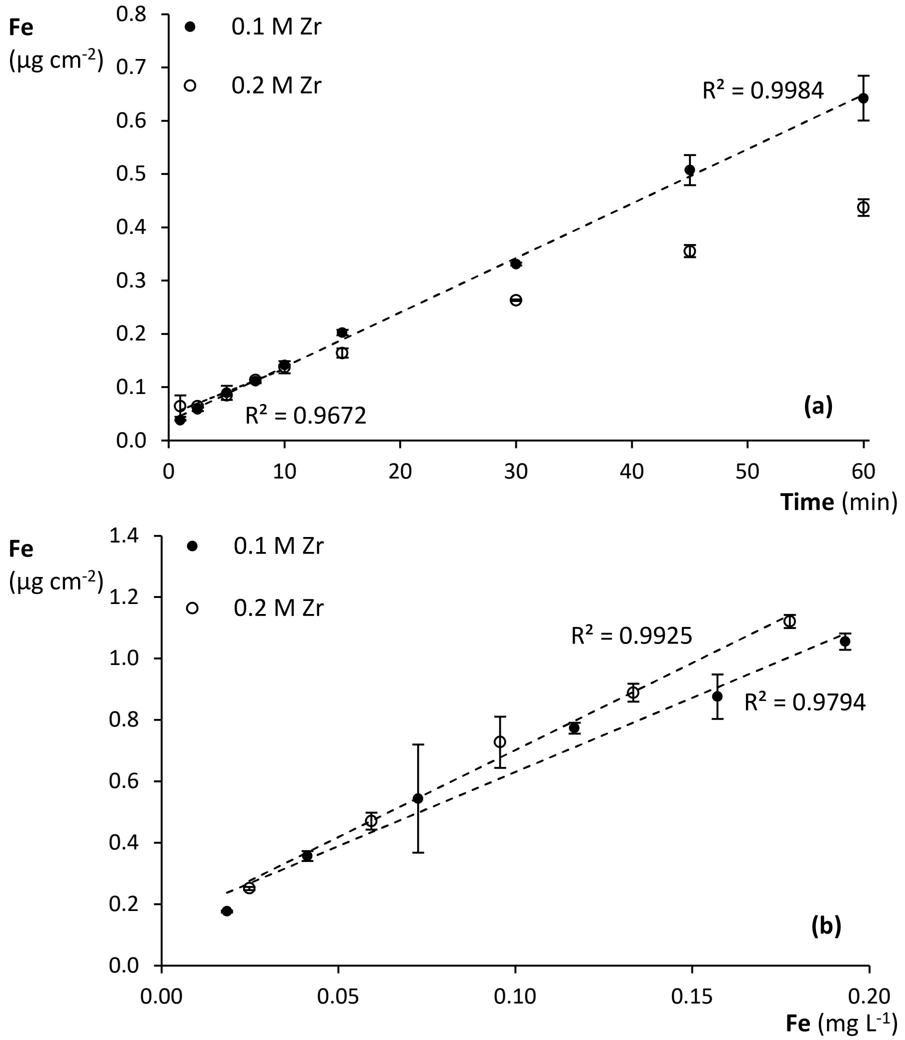

Figure S10. Linear uptake of Fe-NOM colloids (µg Fe cm^{-2}) in 0.1 and 0.2 M $ZrOCl_2$-based binding gels as a function of **(a)** time and **(b)** Fe-NOM concentration in suspension (Figure 5). The gel discs were (a) simultaneously deployed in a Fe-NOM suspension (0.5 mg Fe L^{-1}) between 1 min and 24 h, and (b) individually deployed in DGT housings in Fe-NOM suspensions with 0.05-5 mg Fe L^{-1} initial concentrations for 24 h. The error bars represent standard deviations (n = 2).

S2.3. Summarizing Table

Table S6. Overview of the accumulated analytes in binding layers (n = 2) tested for sorption of phosphate (PO_4) and colloids of Fe-NOM complexes (Fe-NOM) and NOM-coated hydrous ferric oxides (HFO).

**Binding gel**		**Metal concentration**	**Analyte**		
Hydrogel	Metal precursor	Measured (M)	PO_4 (µg P cm^{-2})	Fe-NOM (µg Fe cm^{-2})	HFO (µg Fe cm^{-2})
DGT Research		0.08	83	3.0	
APA	$ZrOCl_2$	0.05	65	3.3	
APA	$ZrOCl_2$	0.17	150	6.1	
APA	$ZrOCl_2$	0.25	220	4.2	
APA	$ZrOCl_2$	0.39	290	2.4	
APA	$ZrCl_4$	0.14	100	4.6	
APA	$ZrCl_4$	0.26	200		
APA	$Zr(OC_4H_9)_4$	0.19	440	1.3	
APA	$Zr(OC_4H_9)_4$	0.29	500		
APA	$TiCl_4$	0.10	67	5.5	
APA	$TiCl_4$	0.36	160		
APA	$Ti(OC_4H_9)_4$	0.08	68	3.7	
APA	$Ti(OC_4H_9)_4$	0.39	180		
APA	$NbCl_5$	0.26	120	2.5	
APA	$NbCl_5$	0.30	160		
APA	$Nb(OC_4H_9)_5$	0.17	100	4.7	
APA	$Nb(OC_4H_9)_5$	0.27	140		
DGT Research		0.08		5.4	3.8
APA				1.2	1.6
APA	$ZrOCl_2$	0.14		11	6.7
APA-PVP				1.8	3.1
APA-PVP	$ZrOCl_2$	0.15		12	8.7
Agarose				0.2	2.7
Agarose	$ZrOCl_2$	0.09		14	7.4
Agarose-PVP				0.3	2.8
Agarose-PVP	$ZrOCl_2$	0.09		12	8.8

REFERENCES

(1) Zhang, Hao.; Davison, William. Performance Characteristics of Diffusion Gradients in Thin Films for the in Situ Measurement of Trace Metals in Aqueous Solution. *Anal. Chem.* **1995**, *67* (19), 3391–3400. https://doi.org/10.1021/ac00115a005.

(2) Luo, J.; Zhang, H.; Santner, J.; Davison, W. Performance Characteristics of Diffusive Gradients in Thin Films Equipped with a Binding Gel Layer Containing Precipitated Ferrihydrite for Measuring Arsenic(V), Selenium(VI), Vanadium(V), and Antimony(V). *Anal. Chem.* **2010**, *82* (21), 8903–8909. https://doi.org/10.1021/ac101676w.

(3) Zhang, H.; Davison, W. Diffusional Characteristics of Hydrogels Used in DGT and DET Techniques. *Anal Chim Acta* **1999**, *398* (2–3), 329–340. https://doi.org/10.1016/S0003-2670(99)00458-4.

(4) Moens, C.; Waegeneers, N.; Fritzsche, A.; Nobels, P.; Smolders, E. A Systematic Evaluation of Flow Field Flow Fractionation and Single-Particle ICP-MS to Obtain the Size Distribution of Organo-Mineral Iron Oxyhydroxide Colloids. *J. Chromatogr. A* **2019**, *1599*, 203–214. https://doi.org/10.1016/j.chroma.2019.04.032.

(5) Moens, C.; Smolders, E. Suwannee River Natural Organic Matter Concentrations Affect the Size and Phosphate Uptake of Colloids Formed by Iron Oxidation. *Geochim. Cosmochim. Acta.* **2021**, *312*, 375–391. https://doi.org/10.1016/j.gca.2021.07.028.

(6) Pouran, H. M.; Martin, F. L.; Zhang, H. Measurement of ZnO Nanoparticles Using Diffusive Gradients in Thin Films: Binding and Diffusional Characteristics. *Anal. Chem.* **2014**, *86* (12), 5906–5913. https://doi.org/10.1021/ac500730s.
